# Perspectives of Adolescent Girls and Young Women on Optimizing Youth-Friendly HIV and Sexual and Reproductive Health Care in Zambia

**DOI:** 10.3389/fgwh.2021.723620

**Published:** 2021-10-25

**Authors:** Patrick V. Edwards, Sarah T. Roberts, Nachela Chelwa, Lyson Phiri, Laura Nyblade, Drosin Mulenga, Caila Brander, Maurice Musheke, Michael Mbizvo, Sujha Subramanian

**Affiliations:** ^1^Comprehensive Health Research Division, RTI International, Research Triangle Park, NC, United States; ^2^Women's Global Health Imperative, RTI International, Berkeley, CA, United States; ^3^Population Council, Lusaka, Zambia; ^4^Global Health Division, RTI International, Washington, DC, United States

**Keywords:** adolescent girls and young women, HIV, Zambia, youth-friendly services, integrated care, engagement in care

## Abstract

Youth-friendly health care delivery models are needed to address the complex health care needs of adolescent girls and young women (AGYW). The aim of this study is to explore the lived experiences of AGYW seeking comprehensive HIV and sexual and reproductive health (SRH) care and to elicit their preferences for integrated health care services. We conducted in-depth interviews and focus group discussions in Lusaka, Zambia among 69 AGYW aged 10-20 who were HIV-negative or of unknown status and 40 AGYW aged 16-24 living with HIV. The data were coded through deductive and inductive processes and analyzed thematically using modified World Health Organization (WHO) dimensions of quality for youth-friendly services. AGYW expressed preference for one-stop clinics with integrated services that could provide HIV services along with other services such as pregnancy testing and family planning. AGYW also wanted information on staying healthy and approaches to prevent disease which could be delivered in the community setting such as youth clubs. An integrated clinic should address important attributes to AGYW including short wait time, flexible opening hours, assurance of confidentiality and positive staff attitudes. Youth-friendly, integrated care delivery models that incorporate AGYW preferences may foster linkages to care and improve outcomes among vulnerable AGYW.

## Introduction

Youth-friendly health services that target adolescent girls and young women (AGYW) affected by HIV are urgently needed in sub-Saharan Africa. In Zambia, for instance, ~5% of AGYW aged 15-19 years and 11% of young women aged 20-24 are living with HIV, compared to about 4 and 7% of Zambian boys and young men across the same age groups (1–3). Furthering this gender disparity, AGYW in Zambia face many social and economic challenges, such as unequal gender norms, stigma and the lack of resources that limit their access and continued engagement with sexual and reproductive health (SRH) and HIV services (1, 4). Only 40% of Zambian AGYW living with HIV know their status, of which 78% have initiated antiretroviral therapy (ART), and 78% of AGYW on ART are virally suppressed, which reveals significant gaps in youth-targeted HIV care in Zambia (5).

Prior studies in sub-Saharan Africa attribute low adolescent engagement with HIV clinics to issues including stigma and the fear of privacy loss (6–8). Another recent study documented significant challenges with antenatal care, noting that young pregnant women in Zambia lacked a youth-specific space and experienced poor provider attitudes and long wait time for consultations (9). In response to these challenges and in an effort to achieve the global HIV 95-95-95 targets, the Zambian government established a national adolescent health strategy in 2017 mandating the provision of youth-friendly services and is also encouraging evaluations of new care delivery models for AGYW that support the “Test and Start” approach, which prioritizes improving HIV testing and treatment through enhancing linkages to, and retention in, care (10–14).

Although studies have been conducted on barriers to access related to specific health services, no prior research has reported on experiences from the perspective of AGYW affected by HIV in seeking health care services required to meet competing needs (15–17). AGYW are in a period of transition as they age through adolescence and approach adulthood (18). AGYW affected by HIV not only need HIV-related services but also access to contraception, treatment for sexually transmitted infections, ability to seek preventive care such as Human Papilloma Virus (HPV) vaccination to protect against cervical cancer, and options to receive confidential counseling services (14, 18, 19).

The objective of this study was to assess characteristics of youth-friendly health care services that were important to AGYW to inform development of a clinic-based intervention to increase HIV testing and engagement in care. We explored the lived experiences of AGYW seeking health services in urban Zambia to elicit their preferences about the types of services they wanted and how they should be delivered. Research on AGYW preferences is needed to optimize community-clinic linkages to empower AGYW engagement with health care services. The findings from this study can be used to develop comprehensive health care delivery models, specifically targeting vulnerable AGYW to improve initiation and retention in care.

## Methods

A qualitative study was conducted in early 2019 consisting of in-depth interviews (IDIs) and focus group discussions (FGDs) with AGYW to elicit preferences for health care delivery including integrated provision of multiple services. This study included AGYW who are HIV-negative or of unknown status (HIV-/u AGYW) along with AGYW living with HIV (YLHIV) in Lusaka, Zambia.

### Participant Eligibility and Recruitment

All participants had to be female and fluent in English, Nyanja, or Bemba, and not pregnant by self-report. HIV-/u AGYW had to be ages 10-20 (inclusive) and must have self-reported their HIV status as negative or unknown. YLHIV had to be ages 16-24 years (inclusive) and diagnosed with HIV within 3 years prior to participation (to target more recently diagnosed AGYW who may better recall their initial experiences). We selected a younger cohort of HIV-/u AGYW than the YLHIV so we could target girls who would be the ideal cohort to receive services related to HIV prevention. The rate of HIV infection increases substantially as this cohort of adolescent girls age (20). Pregnant AGYW were excluded as they require specialized services for maternal and child health, which was beyond the scope of our study. Sampling was stratified by age group (10-12, 13-15, 16-20 for HIV-/u participants; and 16-20 and 21-24 for YLHIV), with eight IDIs and two FGDs per group (40 IDIs and 10 FGDs total). We stratified by these age groups to capture differences in experiences among AGYW of different developmental stages and to simplify the topics for the youngest age groups.

Participants were purposively selected to maximize variability across target age groups. AGYW were identified from nine Lusaka clinics selected to represent areas of diverse socioeconomic status. HIV-/u participants were recruited based on recommendations from the study's Community Advisory Board (CAB), Youth Advisory Board (YAB), and local public health facility staff. To avoid involuntary disclosure of their HIV status to the research team, YLHIV were recruited by staff at the HIV care clinics. Written permission was obtained for study staff to contact and conduct one-on-one sensitization about the study and invite participants to join.

### Data Collection

IDIs and FGDs were conducted in the preferred language of the participant (English, Bemba or Nyanja), in private settings by one of seven experienced, Zambian, female interviewers using semi-structured guides tailored by age group and HIV status. Topics for HIV-/u participants included HIV knowledge, experiences with SRH services such as HIV testing, access to health services, social and family support, stigma, and feedback on proposed interventions. Topics for YLHIV included HIV diagnosis, engagement in care, ART use, disclosure and support, stigma, and feedback on the proposed interventions. Summaries of each IDI and FGD were documented by interviewers in summary reports within 1 day of the discussion (21). The lead author reviewed each of the summary reports to provide rapid feedback to the interviewers on techniques and probing methods. The study team held debriefing sessions on a regular basis during data collection to provide recommendations and make data collection improvements.

### Ethics

Prior to any study procedures, we obtained written informed consent from all participants aged 18 and above using the participants' preferred language, along with assent and parental consent for participants below age 18. The Ethics and Research (ERES) Converge Institutional Review Board in Zambia and the Institutional Review Boards at the Population Council and the University of North Carolina—Chapel Hill provided ethical approval for this study.

### Analysis

Interviewers audio-recorded all interviews and focus groups, and simultaneously translated and transcribed in English using a standard transcript template. Transcription was done by a staff member who did not conduct the interviews to ensure neutrality and the study coordinator performed quality control checks on all transcripts to ensure the accuracy of transcription and translation.

The study team used deductive and inductive processes to develop a codebook using the IDI/FGD guides and summary reports to identify codes. A team of six researchers used Dedoose (2019) software to code and analyze the qualitative data. Throughout the analysis process, ~15% of the transcripts were used to create coding tests to measure and ensure inter-coder reliability was above 70%. Following each test, coding discrepancies were discussed, reconciled, and the codebook and transcripts were updated to reflect agreements.

This analysis focused on both positive and negative experiences faced by participants in accessing HIV testing, HIV treatment, and SRH services, including family planning, STI screening and treatment, and pregnancy testing. Data from relevant codes were extracted from Dedoose and were summarized into analytical memos by three trained analysts, two of whom led the coding. The study team held regular meetings to review emerging themes and to synthesize findings across memos that related to the AGYWs' clinic experience. Clinic preferences were identified and organized within a modified World Health Organization (WHO) framework for youth-friendly services (22, 23). This framework indicates that services should be: (1) appropriate (the right mix of comprehensive health services needed are provided); (2) accessible (able to obtain health services provided); (3) acceptable (health services meet expectations of adolescents); (4) equitable (all adolescents able to obtain health services needed); and (5) effective (health services that help improve health outcomes). The five dimensions of youth-friendly services, their definitions in the context of AGYW in this research, and selected questions from the interview guides that align with each of the dimensions are outlined in Table 1. We conducted a stratified analysis to explore variation in clinic preferences by AGYW HIV status and also noted any differences by age group. English translations of direct quotations were also excerpted to illustrate the themes that arose and are presented within the results.

**Table 1:** Modified World Health Organization (WHO) framework for youth-friendly service provision and selected questions from interviews and focus groups.

**Element of youth-friendly services**	**Definition in context of adolescent girls and young women (AGYW)**	**Selected questions**
Appropriate	Having the right mix of comprehensive health services that are tailored toward AGYW	Have you ever been to the clinic for SRH services? What services did you seek? Did you receive them?Now, imagine that there was a place in the health facility that was only open to adolescent girls and young women, where you could go for information and services to keep yourself healthy, including HIV testing, family planning, HPV vaccination, and sexual health education. What do you think of this plan? Do you think you would use such a clinic? Would young women be interested in coming to this clinic for the HPV vaccination?
Accessible	Ability of AGYW to reach and obtain health services at clinic	What makes it easy or difficult to visit the clinic?How far is the clinic from your home?How long is the long wait time at the clinic to see a nurse or doctor? What wait time would be acceptable?Do you have any challenges with the clinic opening hours? If so, what hours would be more convenient for you?What will you have to pay for services at the clinic?What about the cost of transport to the clinic?
Acceptable	Having a clinic and service experience that meets the expectations of AGYW	What was your experience like at the clinic?What made you feel comfortable?How did you feel about the clinic staff? How did they receive you?Did you feel that the clinic is “youth-friendly”? That is a place where young people are welcomed by the clinic staff and treated with respect. Why or why not? Is this important to you?How confident do you feel that the clinic staff will keep your health information private? What makes you feel this way?
Equitable	Having equal access to services irrespective of HIV status or age	Has anyone ever judged you or made you feel bad about yourself for getting sexual and reproductive health services?Has anyone ever judged you or made you feel bad about yourself because you are living with HIV?
Effective	Having knowledgeable providers and health services that are designed to improve health outcomes among AGYW	What did you think about the quality of the care you received?Did the nurse or doctor provide good advice or treatment?Who do you talk to when you have questions or concerns related to your sexual or reproductive health?Who do you talk to when you have questions or concerns related to your HIV status or your medication?

*Elements of youth-friendly services are based on World Health Organization quality of care framework; Selected questions are not listed in the order they were asked; AGYW, adolescent girls and young women*.

## Results

**Figure 1:**
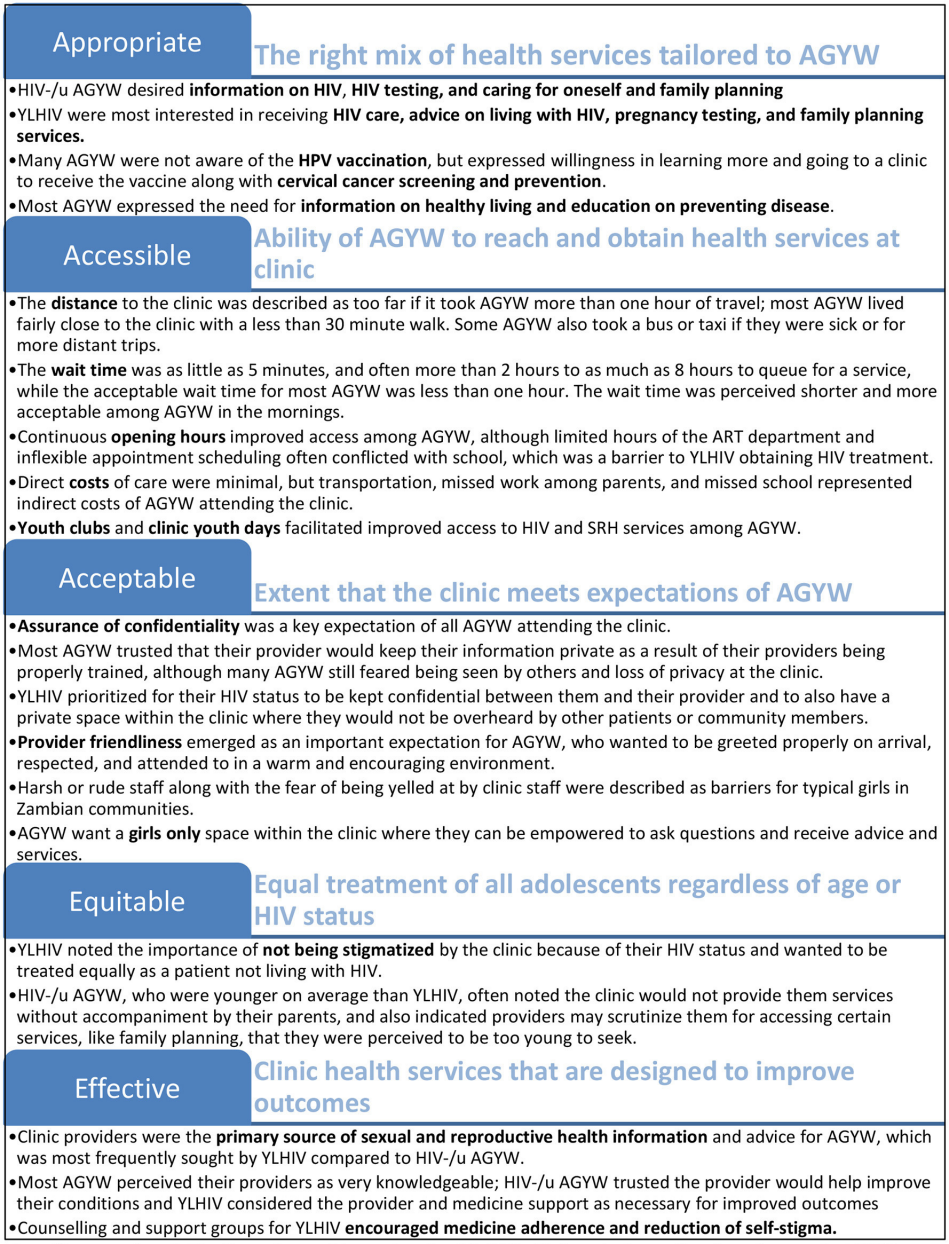
Key attributes identified for youth-friendly service provision among adolescent girls and young women (AGYW) in Zambia. Elements of youth-friendly service provision are based on World Health Organization quality of care framework. AGYW, adolescent girls and young women; ART, anti-retroviral treatment; HIV-/u AGYW, adolescent girls and young women who were HIV-negative or of unknown status; YLHIV, adolescent girls and young women who are living with HIV. Bullets that describe AGYW are findings reflected across both HIV-/u AGYW and YLHIV.

The study enrolled 109 participants, including 69 HIV-/u AGYW (24 IDI and 45 FGD participants) and 40 YLHIV (16 IDI and 24 FGD participants). Participant characteristics are shown in Table 2. The median age was 14 years (interquartile range: 12-16) among HIV-/u participants and 20 years (interquartile range: 17-22) among YLHIV. Very few HIV-/u AGYW reported ever having sex (about 10%) compared to 55% of YLHIV. Only 24 HIV-/u participants (35%) had ever tested for HIV, with about a fourth of the cohort being tested within the past year. Key attributes identified for youth-friendly health services are presented in Figure 1 and complemented by illustrative quotes for selected key attributes (using pseudonyms for participants) in Table 3. Themes relating to clinic preferences were largely common across both HIV-/u AGYW and YLHIV and key differences that emerged between these groups are specified.

**Table 2:** Characteristics of study participants.

	**HIV-/unknown**		**Living with** **HIV (YLHIV)**		**Total**	
	***n***	***%***	***n***	***%***	***n***	***%***
Total	69	100	40	100	109	100
Age (median, IQR)	14	(12–16)	20	(17–22)	16	(13–19)
**Highest level of education**						
None	1	1.45	2	5	3	2.75
Primary school*	38	55.07	10	25	48	44.04
Secondary school*	30	43.47	28	70	58	53.21
Currently in school	56	81.16	15	37.5	71	65.14
**Lives with**						
Parents	55	79.71	20	50	75	68.81
Grandparent	3	4.35	0	0	3	2.75
Other family	20	28.99	17	42.5	37	33.94
Husband or male partner	0	0	3	7.5	3	2.75
Alone	0	0	1	2.5	1	0.92
**Relationship status**						
Single/never married	69	100	37	92.5	106	97.25
Married	0	0	3	7.5	3	2.75
**Source of income**						
None	65	94.2	27	67.5	92	84.4
Informal employment	4	5.8	12	30	16	14.68
Formal employment	0	0	1	2.5	1	0.92
**Other characteristics**						
Ever had sex	7	10.14	22	55.00	29	26.61
**Among HIV-/unknown AGYW**						
Ever tested for HIV	24	34.78	N/A		N/A	
Tested for HIV in past year	18	26.09	N/A		N/A	

* *Primary school and Secondary school include those who have completed at least some school at this level; IQR, Interquartile range; YLHIV, AGYW living with HIV*.

**Table 3:** Illustrative quotes across selected key attributes of youth-friendly service provision.

**Themes**	**Illustrative quotes**
**Appropriate**	
Integration of services	If you want to learn about HIV and AIDS, instead of you going inside[clinic] you can go there [integrated care services] and ask about more information like how can you treat yourself with the right medication and they can answer your question. (Mwiinga, IDI participant, HIV-/u AGYW, age 12)
	There should be pregnancy testers, condoms, morning after pills (Mercy, IDI participant, YLHIV, age 22)
	Because we will find a lot of activities [in an integrated care services clinic] because others fail to find money to buy pregnancy tests so it can be a chance to get them for free there and also there will be a lot of prevention of many diseases because people will have knowledge… [Can prevent diseases] like cervical cancer because of HPV vaccination. (Mapala, IDI participant, YLHIV, age 22)
HPV vaccination and cervical cancer screening	I'm also interested but they only vaccinate those who are 12 years and below (Constance, IDI participant, HIV-/u AGYW, age 18) [NOTE: HPV vaccine are approved for ages 9 to 26 years] [In regards to if a girl would come to the integrated clinic for HPV vaccination]: They can be coming because they will be more comfortable than how they feel when they for to the main clinic, for example if I want to go for cervical cancer screening where adults go and I find my mother they will ask me why went there. (Mwansa, FGD participant, YLHIV, age 21)
SRH services	I bet they would feel comfortable or something because most young girls don't want their parents to be there when they are doing a pregnancy test. (Fridah, IDI Participant, YLHIV, age 21)
	I think you should empower more youths and explain to them about the importance of them knowing or having information on reproductive health. Yes, having access to condoms. (Cynthia, IDI participant, HIV-/u, age 18)
**Accessible**	
Wait time	There is nothing easy because you have to be in the queue when you get there. You wait for people who came earlier than you that is when you get treatment. (Cynthia, IDI participant, HIV-/u, age 18)
Youth programming	We go for a club called youth-friendly corner, we learn about how to do… how to start up a business as an entrepreneur. I love it, because you can even ask about what you don't know. You just feel free there. There are no big people, there no parents, mmm, there people under [same age as herself] the age of you, and so you just feel free. (Jennifer, IDI participant, HIV-/u AGYW, age 15)
**Acceptable**	
Awareness of confidentiality	Yes, and they're not allowed to share information about someone to anyone. It's between me and the doctor. Why? They need to ask for my permission first. I should be the one to tell them, no I will tell them first in my own personal time. (Lubono, IDI participant, HIV-/u AGYW, age 18)
	There should be confidentiality that whatever we discuss remains here and should not go around the community. (Georgina, FGD Participant, YLHIV, age 17)
Need for privacy	It must be a place where, whatever is discussed should not be heard …. (Lynn, FGD Participant, HIV-/u AGYW, age 14)
Provider friendliness	When the leaders are friendly and free, we can as well be open to them. (Chilufya, IDI participant, HIV-/u AGYW, age 17) We are being supported by every department here, they come to check the ART, the sister in charge, and they know all of us by name that this one is seen that one what is wrong, and we give the reason why others are not seen. They are concerned about us. (Lutendwa, IDI participant, YLHIV, age 21)
Girls only	Sometimes there are questions to be asked where only girls are found… this will make them to be free to open up to share what they go through. (Malindi, FGD Participant, YLHIV, age 20)
	Because it is for girls only if you want to test, education on HIV, you just go. …. There will be no boys to see that that one has gone to test. (Victoria, IDI Participant, HIV-/u AGYW, age 15)
**Equitable**	
Equal treatment by HIV status and age	There is this woman at the clinic, when I go to get medicine, she starts shouting at me and saying that I should wait first she attends to children. I tell her that I am also a child then she says no you are an adult and you even know where you got the disease from. I tell her please do not judge me because you do not know how I got this disease. So, every time if that woman is on duty, I do not go there, I go and change my appointment day. (Mwaka, FGD participant, YLHIV, age 20)
	[Going to clinic alone as a young person] Because I also have the right to speak up. I deserve respect, he doesn't need to be rude to me. I know that doctors work hard but needs to be patient when asking somebody. Whenever his being rude to me I have to tell him that no this is not how you are supposed to work. (Lubono, IDI participant, HIV-/u AGYW, age 18)
**Effective**	
Provider knowledge	Yes, she can talk to doctors and nurses because they have the knowledge on such things such as puberty and menstruation. (Dalitso, FGD participant, HIV-/u, age 18)
	Yes, it is possible because at the clinic or health center, they have those people who are specialized in counselling and they can give you more information and answer your questions. (Isubilo, FGD participant, HIV-/u, age 18)

*All names are pseudonyms. FGD, Focus Group Discussion; HIV-/u AGYW, adolescent girls and young women who were HIV-negative or of unknown status; IDI, In-depth Interview; YLHIV, adolescent girls and young women who are living with HIV*.

### Appropriate Service Mix

Both YLHIV and HIV-/u AGYW expressed interest in integrated services that specifically target youth such as HIV testing and treatment, cancer screening, family planning and reproductive health. Currently these services are provided in a vertical manner. For instance, HIV treatment is provided at the antiretroviral therapy (ART) clinic while SRH services are provided at the maternal and child health unit. YLHIV expressed interest in a one-stop place specific for girls to receive multiple services with specific preferences for receiving pregnancy testing and advice related to family planning without having to navigate different service-specific units of the clinic. One YLHIV noted that this structure will make clinic navigation “easier because you do not have to move from this department to other departments” and another YLHIV liked the idea saying that “when they come for other programs, they may want to get tested too.”

Many HIV-/u AGYW and some YLHIV, especially among younger age groups, were not aware of the HPV vaccination but expressed interest in cervical cancer screening and in learning more about receiving the HPV vaccination to reduce their risk of cervical cancer. HPV vaccination and cervical cancer screening was perceived by some AGYW as being only for older women or younger girls, and they were not aware that it was approved in Zambia for ages 9-26 years. When asked directly, most AGYW indicated that they would be willing to go to a clinic to receive the HPV vaccination.

### Access to Services

Many YLHIV and HIV-/u AGYW indicated that they lived close to the clinic (within 30 min walking) and were able to walk or take a quick bus. Those living further away also walked if they could not afford the bus or a taxi. The distance was described as a barrier in instances where it took AGYW longer than 1 h to get to the clinic; however, a few YLHIV worried that living too close to the clinic could expose them to stigma by neighbors who may judge them as “promiscuous” for seeking health services. Similarly, HIV-/u AGYW were also concerned with potential stigma in attending a close health clinic because they felt community members may assume that they are living with HIV, if they are seen seeking health services.

Waiting times varied from 5 min to as high as 8 h across various types of services. YLHIV and HIV-/u AGYW often had to queue more than 2 h, with most considering a wait time more than 1 h as unacceptable, especially during severe illnesses. Many AGYW found shorter queues in the early morning but often blamed long waits and congestion due to staff shortages (especially at drug collection points and at ART sites for YLHIV) along with some staff not arriving on time.

Most AGYW indicated that they had the flexibility to access general services at any time; however, YLHIV indicated that ART departments were not open early enough for them to attend before school or other business. As a result, many YLHIV missed drug collection appointments due to educational commitments and exams.

Direct costs of care among all AGYW were minimal because HIV testing and treatment in Zambia is free. Participants simply paid for the clinic record book, which cost < US $1.00; however, participants paid more if they needed to take a bus or a taxi. HIV-/u AGYW also described higher costs of purchasing medication for other conditions from a pharmacy if it was unavailable at the health facility.

YLHIV and HIV-/u AGYW described positive experiences in their engagement with youth programs that encourage access to services at many clinics, including community-based youth clubs for both genders regardless of HIV status, clinic-based support groups specific for YLHIV, youth-friendly corners led by fellow youth that help adolescents navigate the clinic for services, and clinic youth days that prioritize adolescents and young adults to access services and HIV testing. One younger HIV-/u AGYW liked their clinic's youth-friendly corner describing it as a place with “no big people, no parents… and people under [same age as herself], and so you just feel free.”

### Acceptability of Services

Confidentiality was a key component of acceptability for all AGYW. YLHIV and HIV-/u AGYW largely trusted that their provider would preserve their confidentiality due to their proper training; however, some feared their doctor telling their friends, family, or other clinic patients about their visit. YLHIV prioritized keeping their HIV status private between them and their health provider and having a space within the clinic where they would not be overheard by other patients or community members. Participants expressed concerns with privacy during queueing, with HIV-/u AGYW not wanting others to assume they were seeking HIV care, and YLHIV fearing public disclosure of their status, such as through use of differently colored cards that could identify them as a YLHIV. Highlighting the need for privacy, one HIV-/u AGYW exclaimed “When I just reach the clinic, I feel scared that maybe I can meet someone I know or someone that I live close with because they can start telling other people in the community that I am HIV positive.” Both YLHIV and HIV-/u AGYW also described the need for a girls-only space where they could be empowered to ask questions and receive advice in a private setting separate from boys, young men, and older adults.

YLHIV and HIV-/u AGYW also emphasized the importance of having friendly providers and clinic staff, which helped them to feel respected and cared for when visiting the clinic. Some AGYW described negative experiences attending the clinic, such as having harsh providers, being yelled at or judged by clinic staff for their attendance or having providers who would show up late to the clinic. One AGYW even noted that she would avoid going to the clinic or would switch her appointment day if a rude provider was on duty. Despite the negative experiences, all AGYW wanted to be received with respect despite their young age and to be empathetically engaged on arrival, be tended to while waiting, such as being given blankets and water, be prioritized for faster treatment when ill, and receive compassionate care such as being encouraged during treatment. HIV-/u AGYW, who were mostly younger than YLHIV, often expressed shyness when communicating with their provider but felt more open and confident to answer questions when they had welcoming staff.

### Equitable Care Among AGYW

AGYW wanted to be treated equally regardless of their age or their HIV status. Many participants in the HIV-/u cohort were of young adolescent age and lacked experience seeking SRH care. The youngest participants often described that they cannot go to the clinic unaccompanied and also feared that they would be treated poorly for being too young to access HIV or SRH-related services, which was a direct contrast to the way older or married AGYW was treated. However, some young participants speculated that they would be treated well regardless of if they went alone. One HIV-/u AGYW described that they are confident going to a clinic alone as a young person “because I also have the right to speak up… I deserve respect.” This AGYW described the need for girls to advocate for themselves and that providers should be patient and respectful of patients of younger ages. YLHIV also noted the importance of staff not stigmatizing patients for their HIV status but treating them equally to patients who are not living with HIV.

### Effectiveness of Services

Efficient and high-quality medical care also affirmed AGYW seeking care. AGYW expressed general satisfaction with the medical services along with the advice received by providers. Receiving a remedy to their ailment and feeling better due to the treatment received was important to AGYW. Thus, from the perspective of several participants, feeling that their medical issues could be resolved was a facilitator to their willingness to return to the clinic in the future. AGYW described their doctors as their key source of information and support on SRH, which was especially sought by YLHIV. Providers were described by HIV-/u AGYW as knowledgeable on general health conditions and healthy lifestyles while YLHIV saw providers as knowledgeable on topics of pregnancy prevention, childcare and breastfeeding, STI prevention, and in preventing HIV transmission between them and their partner. Both HIV-/u AGYW and YLHIV trusted their doctor to give them the right advice and relied on this advice to improve their health conditions. YLHIV noted the importance of initial counseling along with support groups to educate, promote self-acceptance and to establish a routine for ART adherence, factors which were considered crucial to managing their HIV status.

## Discussion

This study identified several defining attributes of a youth-friendly, integrated health care delivery model based on clinic experiences and preferences of AGYWs in Lusaka, Zambia. These included (1) having a one-stop location with bundled services applicable to AGYW that includes HIV and SRH services, advice on healthy living and disease prevention; (2) ability to promptly access services with flxibility around other commitments, such as school; (3) provision of services in a private location (preference for clinic dedicated just for girls) with assurances of confidentiality at all points of contact with clinic staff and providers; (4) friendly staff that respect and empower all AGYW; and 5) knowledgeable providers (including staff, counselors, and youth program leaders) that encourage sustained AGYW engagement to improve health outcomes over time. These findings, when aligned to the WHO's dimensions of quality, identify specific attributes that should be prioritized in the design of a youth-friendly, integrated service delivery model to improve engagement in care among AGYW.

HIV-affected AGYW reveal a strong preference for integrated services that include comprehensive SRH services. This study confirms findings from other studies and adds experiences specific to the Zambian context that highlight the barriers among AGYW seeking pregnancy testing, contraceptive services and treatment for sexually transmitted infections (17, 24). Integrating care through access to a comprehensive set of health services from a single location in the clinic could help to address privacy and confidentiality concerns by reducing instances where AGYW have to access individual services separately for HIV care, SRH needs and other health information (15, 25). Additionally, ensuring that staff are friendly and are well-trained can encourage AGYW to use integrated services. In a study to assess the effects of various integrated care models on service uptake among AGYW in Malawi, integrated youth-friendly spaces that expanded opening times and included specialized stigma-reduction training among staff significantly increased the uptake of HIV testing, condoms, hormonal contraception, and STI services compared to the standard of care (25).

The HPV vaccination, which is generally not stigmatized as it is usually provided before sexual debut, can serve as an entry-point to provide expanded selection of services to AGYW (19). Our study highlights the willingness Zambian AGYW have in receiving the HPV vaccination with other HIV services along with the need for AGYW, especially among younger age groups, to have the educational and clinic resources needed for cervical cancer screening and prevention. These self-reported preferences were validated through a pilot test exercise conducted by the study team in which 25 AGYW chose to receive the HPV vaccination and provided positive feedback on the integrated clinic services (19). Our study found that the lack of knowledge on age eligibility by some AGYW suggests a need to offer these services in AGYW-specific settings. Women in sub-Saharan Africa face a dual burden of HIV and cervical cancer and the use of HPV vaccination provides an opportunity to substantially reduce mortality and morbidity (26). The findings from this study contributes new information to the literature on preferences for appropriate clinic services for youth by integrating HIV care with the provision of the HPV vaccination and cervical cancer screening and prevention.

Among other attributes related to access, the most significant barrier identified was the waiting time. Consistent with studies in South Africa and Zimbabwe that attributed long wait times to staffing shortages, AGYW in Lusaka often waited over 2 h and as high as 8 h to see a provider (15, 27). AGYW were often forced to choose between missing school, work, or other productive activities and missing clinic appointments. As younger AGYW were typically accompanied by a parent or older family member, long waits may also result in missed employment for their parent or family member as well. This study shows that wait time represents a substantial indirect cost for many Zambian AGYW, which could result in a larger burden to families than the direct cost of care. This finding is of particular importance to designing a targeted intervention in the urban Zambian context, as addressing this barrier to access could yield a stream of improvements across AGYWs' educational and economic matters along with improved uptake and engagement in health services, especially among the most vulnerable youth. Youth-friendly service delivery models should incorporate approaches to minimize wait time.

Several limitations of this study should be noted. As HIV-/u AGYW were recruited based on advisory board and clinic leadership recommendations, and YLHIV by staff based at the clinics, this study could be subject to selection bias in that those who participated were likely already more engaged in care than AGYW who did not participate. As such, our study may fail to capture barriers faced broadly by AGYW and especially by the most vulnerable AGYW who are not engaged along the HIV care continuum. Another study limitation was the limited level of detail captured in interviews and focus groups from the HIV-/u AGYW due to their limited experiences. While many themes were reflected across both HIV-/u AGYW and YLHIV, many preferences were described and portrayed in a higher level of detail and expression among the YLHIV. We did not ask about clinic preferences specific to more vulnerable or marginalized adolescents, including LGBTQ youth, due to the generalized nature of the HIV epidemic in Zambia. However, interventions designed to enhance engagement in care should especially incorporate youth-friendly services for all adolescents by ensuring the provision of equitable care, confidentiality, and stigma-reduction training among staff. Despite these limitations, this study highlights important barriers and facilitators that are common to and distinct between HIV-/u AGYW and YLHIV and contributes to our understanding of what makes services youth-friendly from the perspective of AGYW.

Our study is one of the first to report on the lived experiences of both YLHIV and HIV-/u AGYW as they seek a range of health services, including the HPV vaccination. This research identifies preferences among younger and older Zambian YLHIV along with HIV-/u AGYW in accessing and engaging in HIV and sexual and reproductive health services. In support of efforts to meet the global 95-95-95 targets, tailored clinical services should incorporate feedback and preferences from AGYW across diverse communities to optimize their youth friendliness and integrate multiple services in one setting, and thereby encourage increased engagement by AGYW across the HIV care continuum.

## Data Availability Statement

The datasets presented in this article are not readily available. The de-identified data set will be stored in a repository and made available in the future in accordance with the NIH Data Sharing Policy found at https://grants.nih.gov/grants/policy/data_sharing/data_sharing_guidance.htm. Requests to access the datasets should be directed to https://grants.nih.gov/grants/policy/data_sharing/data_sharing_guidance.htm.

## Ethics Statement

The studies involving human participants were reviewed and approved by the Ethics and Research (ERES) Converge Institutional Review Board in Zambia. Population Council IRB and University of North Carolina at Chapel Hill IRB as well. Written informed consent to participate in this study was provided by the participants' legal guardian/next of kin.

## Author Contributions

PE served as lead author and led analysis, interpretation of results, writing, table/figure creation, editing, and finalization of manuscript. SR served as co-author and contributed to study design, led data collection, training, guided analysis and contributed to interpretation of results, writing, editing, and final review. NC and LP served as co-author and contributed to data collection, analysis, writing, and final review. LN served as co-author and contributed to study design, interpretation of results, manuscript revisions, and final review. DM served as co-author and contributed to data collection, analysis, writing, and final review. CB served as co-author and contributed to analysis, writing, and final review. MM served as co-author and contributed to study design, oversight of data collection, editing, final review, and interpretation of results and writing. SS served as co-author and led study design, contributed to data analysis, interpretation, writing, editing, and final review. All authors contributed to the article and approved the submitted version.

## Funding

This work was supported by the Eunice Kennedy Shriver National Institute of Child Health and Human Development of the National Institutes of Health under Grant Number UG3 HD096908-01.

## Author Disclaimer

The content is solely the responsibility of the authors and does not necessarily represent the official views of the National Institutes of Health.

## Conflict of Interest

The authors declare that the research was conducted in the absence of any commercial or financial relationships that could be construed as a potential conflict of interest.

## Publisher's Note

All claims expressed in this article are solely those of the authors and do not necessarily represent those of their affiliated organizations, or those of the publisher, the editors and the reviewers. Any product that may be evaluated in this article, or claim that may be made by its manufacturer, is not guaranteed or endorsed by the publisher.
